# The Formation of Glycolamide on Interstellar Ices

**DOI:** 10.1021/acscentsci.6c00183

**Published:** 2026-02-24

**Authors:** Serena Viti

**Affiliations:** † Leiden Observatory, Leiden University, PO Box 9513, 2300 RA Leiden, The Netherlands; ‡ Transdisciplinary Research Area (TRA) ‘Matter’/Argelander-Institut für Astronomie, University of Bonn, D-53121 Bonn, Germany

## Abstract

Glycolamide, a precursor to amino acids, is synthesized efficiently at cryogenic temperatures on interstellar nanoparticles through radical coupling mechanisms.

## Graphical abstract

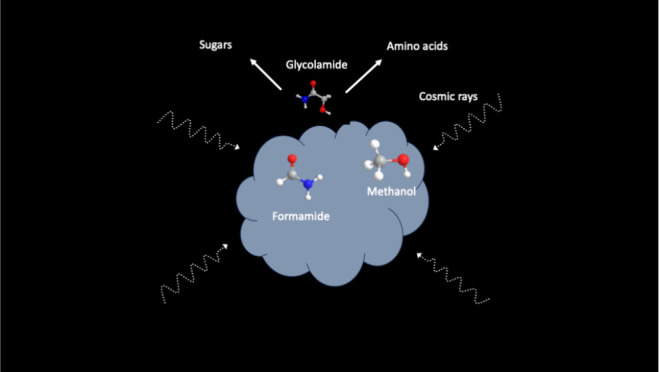

For decades, scientists have searched interstellar space for the chemical seeds of life. In this issue of *ACS Central Science*, Kaiser and co-workers^1^ offer a striking advance in that search by demonstrating for the first time how glycolamide, a molecule closely related to amino acids, can form efficiently under the harsh and cold conditions of the interstellar medium (ISM).

Despite decades of effort, glycine, the simplest amino acid and a fundamental building block of proteins, has remained undetected in space. This absence has puzzled astrochemists because glycine has been detected in meteorites, comets, and even asteroid samples returned to Earth.^2–5^ This paradox sets the stage for Kaiser’s research. Glycolamide is a small organic molecule that has recently been detected in the ISM.^6^ While glycolamide is not itself an amino acid, it is in fact structurally related to glycine. The authors outline a chemical family tree where glycolamide serves as a parent to even more biologically significant molecules. Through various reactions, it can transform into glycine (the missing amino acid), glycolic acid, and other compounds essential for life. This implies that the “missing” glycine might simply be locked away in ices or constantly evolving from precursors like glycolamide. Kaiser and co-workers’ study focuses on the chemical pathways that might lead to glycolamide. They tackle the investigation by combining experimental and theoretical approaches. In the laboratory they recreate key features of the ISM. They deposited thin icy films of simple, well-known interstellar molecules—formamide and methanol—onto cold surfaces of just a few degrees above absolute zero. These ices were then irradiated with energetic electrons that mimic the secondary particles produced by galactic cosmic rays. The authors performed theoretical calculations to help interpret their experimental results. Over time, the experiments revealed that glycolamide forms efficiently through radical chemistry inside these ices.

Their finding is important because it challenges an intuitive assumption about chemistry: that complex molecules require warmth, liquid solvents, or long reaction times. In contrast, the authors show that chemistry in space can be relatively fast, efficient, and surprisingly productive, even at temperatures near 5 K.

They find that on ices, cooled at temperatures down to 5–10 K, the dominant chemical pathway is a recombination of two radicals, both involving carbon, which happens over the lifetime of a typical dense cloud, ∼10^6^ years.

This suggests that molecular complexity can arise early in the life of a molecular cloud, well before stars and planets form, and that some of life’s essential molecular precursors may assemble not on planets but long before planets even exist.

A second key insight of Kaiser and co-workers’ work lies in the experimental mechanism they investigate. The reactions described are “non-equilibrium” processes, driven by energetic radiation rather than slow thermal diffusion. Cosmic rays break simple molecules, creating highly reactive radicals. When these radicals encounter one another in the right orientation, they recombine without an energy barrier (Figure 1). In this case, two carbon-centered radicals join to form a new carbon–carbon bond—one of the most important steps in building biological molecules.

**Figure 1:**
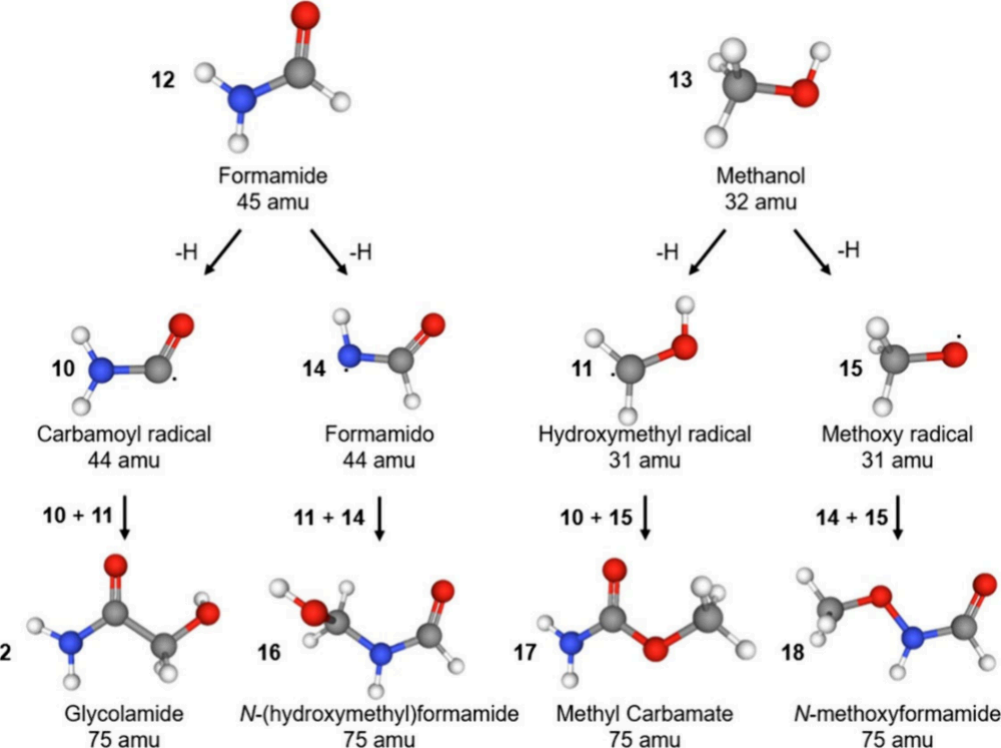
Reaction scheme leading to the first-generation products of formamide-methanol ices at 5 K. Carbon atoms are colored gray, hydrogens are colored white, oxygens are colored red, and nitrogens are colored blue. Reproduced with permission from ref 1. Available under a CC-BY 4.0 license. Copyright 2026 Alexandre Bergantini, Jia Wang, Ivan Antonov, Evgenia A. Batrakova, Sergey O. Tuchin, Ralf I. Kaiser.

The significance of this point is that glycolamide can therefore form rapidly in the ISM from the first-generation radicals carbamoyl and hydroxymethyl.

We do not need to wait for a planet to form and cool down before the chemistry of life can start. Instead, the complex organic molecules required for biology are being synthesized on interstellar dust long before a star even ignites. In other words, space provides both the raw materials (radicals) and the energy needed (via cosmic rays) to assemble prebiotic molecules, and, within the lifespan of a dense molecular cloud, significant amounts of glycolamide may build up on dust grains. Compounds such as glycolamide are plausible precursors to complex amino acids, peptides, and sugars and, once formed in the solid phase, can be released during the early stages of planetary system formation where they may be incorporated into planetesimals, seeding nascent planets with their first complex organic molecules.

This study addresses a question of universal human interest—where do the ingredients of life come from—and sits at the intersection of chemistry, physics, astronomy, and astrobiology. It relies on sophisticated laboratory instrumentation, quantum chemical calculations, and constraints from astronomical observations. Using advanced detection methods—specifically tunable photoionization reflectron time-of-flight mass spectrometry—the team watched this chemistry happen in real-time as they slowly warmed the ice.

Of course, it should be underlined that glycolamide is not glycine, and forming a precursor is not the same as forming life. The experiments are carefully controlled and simplified compared to the true complexity of interstellar ices. For example, the composition of the laboratory sample is formamide/methanol ∼1:1, which most likely does not coincide with the abundances of these species in the ISM. Still, as a proof of concept, the work is very compelling. It demonstrates that, provided there are considerable quantities of formamide and methanol, glycolamide can easily form in cold ISM astrophysical environments.

In conclusion, this study can be considered a reorientation of perspective. It suggests that the precursors of amino acids can be formed on the ices and released to the gas during the very early stages of planet formation, when they can contribute to the material that forms such planets.

## References

[1] Bergantini A., Wang J., Antonov I., Batrakova E. A., Tuchin S. O., Kaiser R. I. (2025). Nonequilibrium Synthesis of Glycolamide
(NH2COCH2OH), a Precursor to Amino Acids, on Interstellar Nanoparticles. ACS Central Science..

[2] Cronin J. R., Moore C. B. (1971). Amino acid analyses
of the Murchison, Murray, and Allende
carbonaceous chondrites. Science.

[3] Koga T., Naraoka H. (2017). A new family
of extraterrestrial amino acids in the
Murchison meteorite. Sci. Rep..

[4] Elsila J. E., Glavin D. P., Dworkin J. P. (2009). Cometary
glycine detected in samples
returned by Stardust. Meteoritics & Planetary
Science.

[5] Altwegg K., Balsiger H., Berthelier J. J., Bieler A., Calmonte U., Fuselier S. A., Goesmann F., Gasc S., Gombosi T. I., Le Roy L. (2017). Organics in comet 67P–a
first comparative analysis
of mass spectra from ROSINA-DFMS, COSAC and Ptolemy. Mon. Not. R. Astron. Soc..

[6] Rivilla V. M., Sanz-Novo M., Jiménez-Serra I., Martín-Pintado J., Colzi L., Zeng S., Megías A., López-Gallifa Á., Martínez-Henares A., Massalkhi S. (2023). First glycine
isomer detected in the interstellar medium:
glycolamide (NH2C­(O)­CH2OH). Astrophysical Journal
Letters.

